# Smoking accelerates aging of the small airway epithelium

**DOI:** 10.1186/s12931-014-0094-1

**Published:** 2014-09-24

**Authors:** Matthew S Walters, Bishnu P De, Jacqueline Salit, Lauren J Buro-Auriemma, Timothy Wilson, Allison M Rogalski, Lindsay Lief, Neil R Hackett, Michelle R Staudt, Ann E Tilley, Ben-Gary Harvey, Robert J Kaner, Jason G Mezey, Beth Ashbridge, Malcolm A S Moore, Ronald G Crystal

**Affiliations:** Department of Genetic Medicine, Weill Cornell Medical College, 1300 York Avenue, Box 164, New York, New York 10065 USA; Cell Biology Program, Memorial Sloan-Kettering Cancer Center, New York, New York USA

**Keywords:** Aging, Small airway epithelium, Telomere, Smoking

## Abstract

**Background:**

Aging involves multiple biologically complex processes characterized by a decline in cellular homeostasis over time leading to a loss and impairment of physiological integrity and function. Specific cellular hallmarks of aging include abnormal gene expression patterns, shortened telomeres and associated biological dysfunction. Like all organs, the lung demonstrates both physiological and structural changes with age that result in a progressive decrease in lung function in healthy individuals. Cigarette smoking accelerates lung function decline over time, suggesting smoking accelerates aging of the lung. Based on this data, we hypothesized that cigarette smoking accelerates the aging of the small airway epithelium, the cells that take the initial brunt of inhaled toxins from the cigarette smoke and one of the primary sites of pathology associated with cigarette smoking.

**Methods:**

Using the sensitive molecular parameters of aging-related gene expression and telomere length, the aging process of the small airway epithelium was assessed in age matched healthy nonsmokers and healthy smokers with no physical manifestation of lung disease or abnormalities in lung function.

**Results:**

Analysis of a 73 gene aging signature demonstrated that smoking significantly dysregulates 18 aging-related genes in the small airway epithelium. In an independent cohort of male subjects, smoking significantly reduced telomere length in the small airway epithelium of smokers by 14% compared to nonsmokers.

**Conclusion:**

These data provide biologic evidence that smoking accelerates aging of the small airway epithelium.

**Electronic supplementary material:**

The online version of this article (doi:10.1186/s12931-014-0094-1) contains supplementary material, which is available to authorized users.

## Introduction

Aging is a complex natural process that affects all living organisms, and can be broadly defined as a progressive decline in homeostasis of biological processes that leads to a loss and impairment of physiological integrity and function with a subsequent increased risk of disease and death [1,2]. The biologic mechanisms of aging are only partly understood, but include genomic instability, telomere attrition, cellular senescence, epigenetic alterations, loss of protein homeostasis (proteostasis), dysregulated nutrient sensing, mitochondrial dysfunction, altered intercellular communication and stem cell exhaustion [1,2]. Similar to other organs, the lung demonstrates a progressive decrease in lung function in healthy individuals [3,4]. Epidemiological studies have demonstrated that smoking results in accelerated lung function decline over time, suggesting smoking accelerates aging of the lung [3,5]. In support of this concept, telomere length analysis of blood leukocytes has demonstrated that smoking is associated with shortened telomeres [6-18].

Based on the data indicating that smoking accelerates the aging-related decline in lung function, and that smokers have shorter telomere lengths in blood leukocytes, we hypothesized that cigarette smoking accelerates aging of the small airway epithelium, the cell population directly exposed to the inhaled toxins from the cigarette smoke, and one of the primary sites in the lung to develop histologic abnormalities associated with cigarette smoking [19-25]. To assess the effect of cigarette smoking on small airway epithelium aging, we compared age matched healthy nonsmokers and healthy smokers with no clinical manifestation of lung disease using the sensitive biologic parameters of gene expression and telomere length. Analysis of small airway epithelium gene expression levels demonstrated that smoking significantly dysregulates expression of an aging gene signature [26]. Further, telomere length analysis in an independent cohort of age matched male subjects revealed that smoking significantly reduced telomere length in the small airway epithelium. Overall, these data provide biologic evidence that before the manifestation of lung function decline, smoking accelerates aging of the small airway epithelium by dysregulation of age-related gene expression and enhanced telomere erosion.

## Methods

### Study population

Healthy nonsmokers and healthy smokers were recruited from the general population in New York City. Individuals were evaluated at the Weill Cornell and Rockefeller University NIH Clinical and Translational Science Centers and the Department of Genetic Medicine Clinical Research Facility, using Institutional Review Board-approved clinical protocols and signed informed consent prior to any procedures. The criteria for “healthy” was based on history, physical exam, complete blood count, coagulation studies, liver function tests, urine studies, chest X-ray, EKG and pulmonary function tests (for full inclusion/exclusion criteria, see Additional file 1, Methods) [25,27,28]. All subjects were negative for HIV1 and had normal α1-antitrypsin levels. Smoking status was verified by urine nicotine and cotinine levels. Nonsmokers were defined as self-reported life-long nonsmokers, with non-detectable urine nicotine (<2 ng/ml) and cotinine (<5 ng/ml); and smokers were defined as self-reported current smokers with urine nicotine ≥30 ng/ml and/or urine cotinine ≥50 ng/ml. Two independent age matched cohorts of nonsmokers and smokers were used in this study to analyze the effects of smoking on small airway epithelium age-related gene expression (Table 1) and telomere length (Table 2).

**Table 1:** **Demographics of the study population and biologic samples for analysis of aging signature gene expression in the small airway epithelium** ^**1**^

**Parameter**	**Healthy nonsmokers**	**Healthy smokers**	**p value** ^**6**^
n	29	29	
Gender (M/F)	16/13	22/7	
Age (yr)	38.0 ± 12.4	38.8 ± 10.1	>0.8
Race (B/W/O)^2^	10/8/11	13/8/8	
Smoking history (pack-yr)	0	17.8 ± 8.4	
Urine nicotine (ng/ml)	negative	1657 ± 1527	
Urine cotinine (ng/ml)	negative	1929 ± 1060	
Pulmonary function parameters^3^			
FVC	106.9 ± 12.1	111.1 ± 11.6	>0.17
FEV_1_	106.3 ± 12.2	109.4 ± 13.1	>0.35
FEV_1_/FVC	82.8 ± 4.9	81.0 ± 4.3	>0.13
TLC	99.7 ± 17.3	97.6 ± 14.1	>0.6
DLCO	90.4 ± 9.6	86.0 ± 5.4	<0.04
Body mass index (BMI)	26.4 ± 5.3	27.8 ± 4.8	>0.3
Epithelial cells^4^			
% epithelial cells	98.9 ± 0.9	99.2 ± 0.8	>0.2
% inflammatory cells	1.0 ± 0.8	0.8 ± 0.8	>0.2
Differential cell count^5^			
Ciliated (%)	71.3 ± 5.3	62.9 ± 6.9	<0.00001
Secretory (%)	10.0 ± 4.5	12.3 ± 6.4	>0.12
Basal (%)	9.5 ± 6.6	9.6 ± 6.6	>0.96
Undifferentiated columnar (%)	8.2 ± 4.9	14.5 ± 8.2	<0.001

^1^Data is presented as mean ± standard deviation.

^2^B = Black, W = White, O = Other.

^3^Pulmonary function testing parameters are given as % predicted value with the exception of FEV_1_/FVC, which is reported as % observed; FVC – forced vital capacity, FEV_1_ – forced expiratory volume in 1 sec. FVC, FEV_1_ and FEV_1_/FVC are post-bronchodilator values. TLC – total lung capacity, DLCO – diffusing capacity of the lung for carbon monoxide.

^4^Small airway epithelium.

^5^As % of small airway epithelium recovered.

^6^Smoker vs Nonsmoker comparison by student’s t-test with p < 0.05 being significant.

**Table 2:** **Demographics of the study population and biologic samples for telomere length analysis in the small airway epithelium** ^**1**^

**Parameter**	**Healthy nonsmokers**	**Healthy smokers**	**p value** ^**6**^
n	21	22	
Gender	All male	All male	
Age (yr)	39.0 ± 12.9	43.9 ± 7.6	>0.14
Race (B/W/O)^2^	8/6/7	14/3/5	
Smoking history (pack-yr)	0	22.6 ± 9.0	
Urine nicotine (ng/ml)	negative	1424 ± 1432	
Urine cotinine (ng/ml)	negative	1359 ± 993	
Pulmonary function parameters^3^			
FVC	103.1 ± 10.9	110.4 ± 8.5	<0.03
FEV_1_	105.4 ± 11.1	107.5 ± 8.2	>0.48
FEV_1_/FVC	83.9 ± 4.8	79.1 ± 3.7	<0.002
TLC	102.0 ± 14.6	96.2 ± 12.7	>0.17
DLCO	93.3 ± 11.8	87.2 ± 7.7	>0.05
Body mass index (BMI)	27.8 ± 4.5	26.0 ± 3.9	>0.16
Epithelial cells^4^			
% epithelial cells	97.5 ± 1.4	99.1 ± 0.8	<0.0001
% inflammatory cells	2.5 ± 1.4	0.9 ± 0.8	<0.0001
Differential cell count^5^			
Ciliated (%)	62.3 ± 5.8	57.9 ± 7.1	<0.03
Secretory (%)	11.0 ± 5.1	17.3 ± 6.0	<0.0006
Basal (%)	5.8 ± 4.2	5.6 ± 4.5	>0.97
Undifferentiated columnar (%)	18.4 ± 5.8	18.3 ± 8.6	>0.90

^1^Data is presented as mean ± standard deviation.

^2^B = Black, W = White, O = Other.

^3^Pulmonary function testing parameters are given as % predicted value with the exception of FEV_1_/FVC, which is reported as % observed; FVC – forced vital capacity, FEV_1_ – forced expiratory volume in 1 sec. FVC, FEV_1_ and FEV_1_/FVC are post-bronchodilator values. TLC – total lung capacity, DLCO – diffusing capacity of the lung for carbon monoxide.

^4^Small airway epithelium.

^5^As % of small airway epithelium recovered.

^6^Smoker vs Nonsmoker comparison by student’s ttest with p < 0.05 being significant.

### Small airway epithelial sampling

Small airway epithelium (10th to 12th order) was collected by fiberoptic bronchoscopy by brushing as previously described [25,27,28]. Following withdrawal of the bronchoscope, the cells were dislodged from the brush by flicking the brush tip in 5 ml of ice-cold bronchial epithelium basal medium (BEBM, Lonza, Basel, Switzerland). An aliquot of all airway epithelial samples was used to quantify the total number of cells recovered, and to quantify the percentage of epithelial and inflammatory cells and the proportions of epithelial cell subtypes. Cells from a second aliquot were pelleted for either RNA or DNA extraction for subsequent transcriptome or telomere length analysis, respectively.

### RNA and microarray processing

Pelleted small airway epithelial cells obtained by bronchoscopy were immediately processed for RNA extraction and microarray analysis as previously described [25,27,28]. Total RNA was extracted from the small airway epithelium of 29 nonsmokers and 29 smokers (Table 1) using the TRIzol method (Invitrogen) with subsequent clean-up using the RNeasy MinElute RNA purification kit (Qiagen, Valencia, CA) and stored in RNAsecure (Ambion, Austin, TX) at −80°C. RNA quality was assessed using a Bioanalyzer (Agilent Technologies, Santa Clara, CA) to determine RNA integrity (RIN value) with only samples displaying a RIN >6 included in this study and concentration quantified with a NanoDrop ND-1000 spectrophotometer (NanoDrop Technologies, Wilmington, DE). One μg of total RNA was prepared for microarray genome-wide transcriptome analysis using the 3’IVT Express Kit (Affymetrix, Santa Clara, CA) and samples were submitted to the Genomics Resources Core Facility at Weill Cornell Medical College for hybridization, washing and scanning of the Affymetrix HG U133 Plus 2.0 microarray GeneChips. All HG-U133 Plus 2.0 microarrays were processed according to Affymetrix protocols, hardware and software, including being processed by the Affymetrix fluidics station 450 and hybridization oven 640 and scanned with an Affymetrix Gene Array Scanner 3000 7G. The captured image data from the HG-U133 Plus 2.0 arrays were processed using the MAS5 algorithm in GeneSpring version 7.3 (Affymetrix Microarray Suite Version 5) and normalized per chip to the median expression value of each sample to generate P calls for filtering. Overall microarray quality was verified by the criteria: (i) 3′/5’ ratio for GAPDH ≤3; and (ii) scaling factor ≤10.0. CEL files were processed by Partek Genomics Suite Software version 6.6 (Partek, Inc., St Louis, MO) for quality control, identification of outliers and determination of expression level for all probesets, using the Robust Multi-chip Average (RMA) method with Partek default parameters.

### Microarray data analysis

Genes containing U133 2.0 microarray probesets and an Affymetrix P call in ≥10% of samples were considered to be expressed in the small airway epithelium. To confirm the genome wide transcriptional changes in response to smoking the phenotypes were evaluated in Partek for sources of variation and an ANOVA was performed to assess the effect of smoking on gene expression corrected for all covariates, including age, gender, race and region (small airway epithelium from distal right *vs* distal left lower lobe of the lung). Smoking-dependent differences in expression identified by Students t test and a p < 0.05 (using a Benjamini-Hochberg correction to limit the false discovery rate) were considered to represent a significant difference in expression between groups. Global patterns of expression were examined using a euclidean dissimilarity and average linkage hierarchical cluster analysis. To assess the effect of smoking on aging of the small airway epithelium, we compared healthy smoker *vs* healthy nonsmoker small airway epithelium expression of a 73 gene aging signature identified by de Magalhaes et al. [26] by meta-analysis of age-related gene expression profiles using 27 datasets from mice, rats and humans representing a common signature of aging between different species and tissues. Using criteria described above to define small airway epithelial expression resulted in removal of 6 genes from the analysis with a final aging signature of 67 genes (Additional file 1: Table S1). The phenotypes were evaluated in Partek for sources of variation and an ANOVA was performed to assess the effect of smoking on gene expression corrected for all covariates, including age, gender, race and region (small airway epithelium from distal right *vs* distal left lower lobe of the lung). Smoking-dependent differences in expression identified by Students t test and a p < 0.05 (using a Benjamini-Hochberg correction to limit the false discovery rate) were considered to represent a significant difference in expression between groups. Global patterns of expression of the 67 aging-related genes were examined using principal component analysis (PCA). The raw data and full dataset are publically available at the Gene Expression Omnibus (GEO) site (http://www.ncbi.nlm.nih.gov/geo/), accession number GSE52237.

### Immunohistochemistry

To analyze expression of aging genes in the small airway epithelium at the protein level immunohistochemistry analysis was performed. Commercially available biopsy sections of paraffin embedded normal human bronchus tissue (Cat No: HuFPT111, US Biomax, Inc., Rockville, MD) were purchased for 3 independent donors (Donor 1: 21 yr old female, Donor 2: 16 yr old female and Donor 3: 37 yr old male). The samples were first cleaned in xylene and rehydrated with graded ethanol followed by steaming the samples for 15 min in citrate buffer solution (Thermo Scientific), followed by cooling at 23°C for 20 min to recover antigens and enhance staining. Endogenous peroxidase activity was quenched using 0.3% H_2_O_2_ for 30 min, followed by incubation for 20 min with normal serum matched to the secondary antibody to reduce background staining. Samples were incubated overnight at 4°C with the following primary antibodies all purchased from Abcam (Cambridge, MA) at specific individual concentrations: rabbit polyclonal anti-C3 antibody (ab129945, 0.2 μg/ml), rabbit polyclonal anti-CX3CL1 antibody (ab85034, 0.2 μg/ml), rabbit monoclonal anti-CLU antibody (ab92548, 0.2 μg/ml), rabbit polyclonal anti-LAPTM5 antibody (ab108014, 0.2 μg/ml), rabbit monoclonal anti-MGST1 antibody (ab131059, 1 μg/ml) and rabbit monoclonal anti-SPP1 antibody (ab91655, 0.2 μg/ml). Isotype-matched IgG (Jackson ImmunoResearch Laboratories Inc., West Grove, PA) was used as a negative control. The Vectastain Elite ABC kit and AEC substrate kit (Dako North America Inc., Carpinteria, CA) were used to visualize antibody binding and slides were counterstained with Mayer’s hematoxylin (Polysciences Inc., Warrington, PA) and mounted using faramount mounting medium (Dako North America Inc.). Images were acquired using a Nikon Microphot microscope with a Plan 40 × N.A. 0.70 objective lens and an Olympus DP70 CCD camera.

### Analysis of telomere length

Pelleted small airway epithelial cells obtained by bronchoscopy or blood derived leukocytes were processed for DNA extraction using the Qiagen Puregene kit (Germantown, MD). DNA was extracted from the small airway epithelium of 21 male nonsmokers and 22 male smokers and matched blood derived leukocytes of a subset of 12 nonsmokers and 16 smokers (Table 2) and the telomere length for each sample determined by Southern analysis using the TeloTAGGG telomere length assay kit (Roche Applied Science, Indianapolis, IN). Briefly, 1 μg of purified DNA was digested with the restriction enzymes *HinfI* plus *RsaI* (20 U each) in a 20 μl reaction volume for 2 hr, 37°C. The reaction was stopped by adding gel loading buffer. Digested DNA was separated by electrophoresis on a 0.8% agarose gel in Tris-acetate ethylenediaminetetraacetic acid (EDTA) buffer at 5 V/cm for 4 h and transferred to a nylon membrane by Southern analysis using 3 M NaCl plus 0.3 M sodium citrate buffer pH 7.0 for 16 hr. The transferred DNA was fixed by UV-crosslinking (120 mJ) to the nylon membrane. The terminal restriction fragments (TRFs) were hybridized to a digoxigenin (DIG)-labeled probe specific for telomeric repeats for 1 hr, 42°C, followed by incubation with a DIG-specific antibody covalently coupled to alkaline phosphatase for 30 min, 25°C. Finally, the immobilized telomere probe on the membrane was detected by incubation with CDP-Star, a chemiluminescent substrate, followed by exposure to an X-ray film. The film was then scanned with a densitometer and the mean TRF length was calculated using Image quant TL software (GE Healthcare, Port Washington, NY). Samples were analyzed in an un-blinded fashion; however to minimize the effect of variability between experiments, equal numbers of nonsmoker and smoker samples were run on each gel (see Additional file 1: Figure S1 for representative gels).

### Statistics

Comparison of demographic parameters among groups was performed by 2-tailed Students t test. For the HG-U133 Plus 2.0 gene expression data, an ANOVA was performed to assess the effect of smoking significance on gene expression corrected for all covariates, including age, gender, ethnicity and region. Differences in expression were evaluated by Students t test and a p < 0.05, using a Benjamini-Hochberg correction to limit the false discovery rate, was considered to represent a significant difference in expression between groups. The difference in mean telomere (TRF) length between phenotypes was calculated by 2-tailed Students t test and a p value <0.05 was considered to represent a significant difference in length between groups. Correlations between mean telomere length and age as well as smoking history were done using both univariate and multivariate linear regression and calculated using commercial software (Stata 10.1, StataCorp, College Station, TX). A p < 0.05 was considered to represent a statistically significant correlation.

## Results

### Effect of smoking on aging-related genes in the small airway epithelium

**Figure 1:**
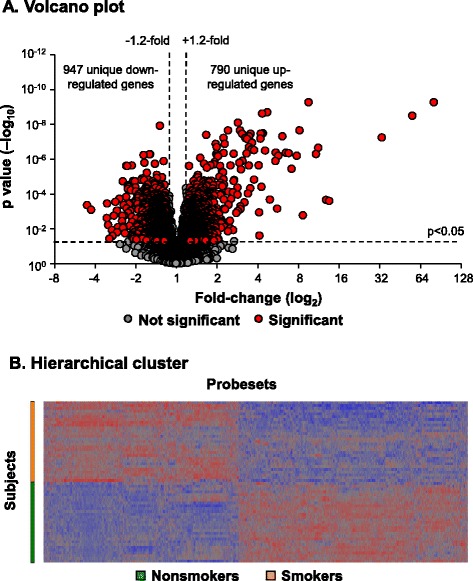
**Smoking dysregulated genes in the small airway epithelium. A**. Volcano plot, smoker *vs* nonsmoker small airway epithelium (n = 29 nonsmokers and n = 29 smokers) using all expressed (P call ≥10) probesets as input dataset. Ordinate – p value; abscissa – fold-change (log_2_). Data demonstrates 2488 smoking-dysregulated probesets [fold-change ≥1.2, p < 0.05 with false discovery rate (FDR) correction] representing a total of 1737 unique genes. **B**. Hierarchical cluster analysis of smoker *vs* nonsmoker small airway epithelium based on expression of 2488 smoking-dysregulated probesets [fold-change ≥1.2, p < 0.05 with false discovery rate (FDR) correction]. Probesets expressed above the average are represented in red, below average in blue and average in grey. The probesets are represented horizontally and individual samples vertically.

To assess the effect of smoking on aging-related gene expression in the small airway epithelium we used a cohort of age matched healthy nonsmokers (n = 29) and healthy smokers (n = 29; Table 1). Cell differential analysis of the small airway epithelial brushes of nonsmokers and smokers demonstrated epithelial cells were the major dominant cell population with a small amount of inflammatory cells. However, no significant differences (p > 0.2) in the total epithelial (nonsmokers, 98.9 ± 0.9% and smokers, 99.2 ± 0.8%) and inflammatory (nonsmokers, 1.0 ± 0.8% and smokers, 0.8 ± 0.8%) cell populations between phenotypes was observed. To confirm the effect of smoking at the mRNA level in our study cohort, we performed genome wide transcriptional analysis to identify the total number of smoking dysregulated transcripts in the small airway epithelium. We hypothesized that small changes in gene expression would indicate the early stages of smoking dependent changes on accelerating aging of the airway/lung. Therefore, we rationalized that a 20% difference in gene expression (i.e., 1.2-fold) would be an acceptable cutoff to robustly detect smoking dependent changes and result in functional biological changes at the protein level for each gene. A volcano plot demonstrated there were 2488 differentially expressed probesets which represent a total of 1737 unique genes (947 down-regulated by smoking and 790 up-regulated by smoking) using criteria of presence of U133 2.0 microarray probesets and a P call criteria of ≥10% of samples, fold-change ≥1.2 and adjusted p < 0.05 with false discovery rate correction for multiple comparisons corrected for all covariates, including age, gender, race and region (distal right *vs* distal left lower lobe; Figure 1A). Unsupervised hierarchical cluster analysis using the 2488 smoking-dysregulated probesets revealed complete separation of smoker and nonsmoker small airway epithelial subjects (Figure 1B).

**Figure 2:**
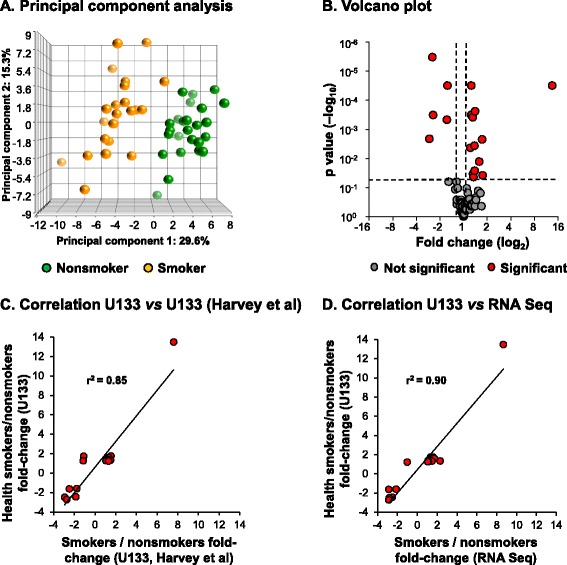
**Expression of an aging gene signature in small airway epithelium of age matched healthy smokers** ***vs*** **healthy nonsmokers. A**. Principal component analysis of gene expression of small airway epithelium of smokers (n = 29, orange circles) and nonsmokers (n = 29, green circles) using the de Magalhaes et al. [26] 67 gene aging signature as an input dataset. Data is corrected for all covariates except smoking status. **B**. Volcano plot, smoker *vs* nonsmoker small airway epithelium of the 67 aging gene signature. Ordinate – p value (log_10_); abscissa – fold-change (log_2_). Red circles represent genes significantly differentially expressed in smoker *vs* nonsmoker small airway epithelium (≥1.2 fold-change up- or down-regulated; p < 0.05 using a Benjamini-Hochberg correction of false discovery rate). **C**. Correlation analysis of the 18 aging genes differentially expressed in the small airway epithelium of healthy smokers *vs* healthy nonsmokers, comparing smoker *vs* nonsmoker fold-changes in U133 gene expression with U133 gene expression from an independent dataset (n = 12 nonsmokers and n = 10 smokers, GSE4498) [25]. **D**. Correlation analysis of the 18 aging genes differentially expressed in the small airway epithelium of healthy smokers *vs* healthy nonsmokers, comparing smoker *vs* nonsmoker fold-changes in U133 gene expression with RNA Seq gene expression from an independent dataset (n = 5 nonsmokers and n = 6 smokers) [27].

**Figure 3:**
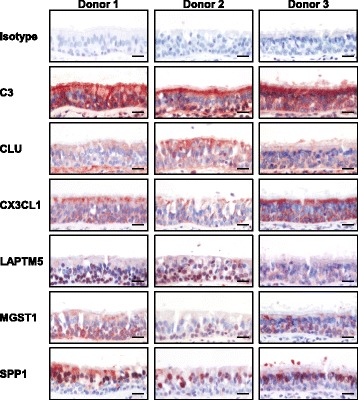
**Immunohistochemical staining analysis of smoking dysregulated aging genes in the small airway epithelium.** Normal nonsmoker human bronchus sections from 3 independent donors were analyzed for expression of C3, clusterin (CLU), CX3CL1, LAPTM5, MGST1 and SPP1 using gene specific antibodies. Isotype specific antibody was used as negative control. Scale bar 20 μm.

To assess the effect of smoking on aging-related gene expression in the small airway epithelium, we analyzed expression levels of a common 73 gene aging signature identified from meta-analysis of age-related gene expression profiles using datasets from mice, rats and humans [26]. Due to the multiple cell types used in the meta-analysis, the aging signature is not specific for epithelial or pulmonary cells *per se*, thus preventing the study of small airway epithelial specific aging genes in response to smoking. However, it likely represents ubiquitously expressed genes that are involved in the natural aging process of all cell types. Based on the presence of U133 2.0 microarray probesets and a P call criteria of ≥10% of samples to be considered expressed, 67 genes of the aging signature were expressed in the small airway epithelium of healthy nonsmokers and smokers (Additional file 1: Table S1). Principal component analysis, using the 67 genes as an input dataset, demonstrated clear separation of the samples by smoking phenotype when corrected for all covariates, including age, gender, race and region (distal right *vs* distal left lower lobe; Figure 2A). A volcano plot identified 13 significantly up-regulated genes and 5 significantly down-regulated genes using criteria of fold-change ≥1.2 and adjusted p < 0.05 with false discovery rate correction for multiple comparisons (Figure 2B). Of the 13 genes significantly up-regulated by smoking, 11 demonstrated the same direction of expression observed with aging (SPP1, LAPTM5, ANXA3, MPEG1, MGST1, MSN, CLU, FCGR2B, GSTA1, LITAF, and VAT1), whereas 2 demonstrated the opposite direction of expression observed with aging (COL3A1 and GHITM; Table 3) [26]. Of the 5 genes significantly down-regulated by smoking, 2 demonstrated the same direction of expression observed with aging (C4A and CX3CL1), whereas 3 demonstrated the opposite direction of expression observed with aging (MT1F, PCSK6 and C3) [26]. As an alternative strategy we assessed expression of the 67 gene aging signature in the list of 1737 unique smoking dysregulated genes identified in our cohort (Figure 1). Using this approach 14/67 (ANXA3, C3, C4A, CLU, CX3CL1, GHITM, GSTA1, LITAF, MGST1, MPEG1, MSN, MT1F, PCSK6 and SPP1) aging signature genes were present in the smoking dysregulated gene list. These 14 aging genes were all present in our original approach which identified 18/67 aging genes to be dysregulated in response smoking. These data demonstrate that independent of the analytic strategy the smoking dependent changes in expression of the aging gene signature are consistent. To validate these gene expression findings, correlation analysis was carried out on the 18 aging genes differentially expressed in the small airway epithelium by comparing smoker *vs* nonsmoker fold-changes in the U133 gene expression with U133 and RNA Seq gene expression data from two independent datasets of age-matched samples [25,27]. The results demonstrate a high degree of correlation in expression pattern of the 18 aging-related genes dysregulated by smoking with both the U133 (r^2^ = 0.85) and RNAseq (r^2^ = 0.90) datasets (Figure 2C-D). Due to the lack of availability of small airway epithelial biopsies from nonsmokers and smokers, we were unable to quantify and assess the smoking dependent mRNA gene expression changes we observed at the protein level. However, to demonstrate epithelial expression of the aging related genes we performed immunohistochemistry staining analysis of commercially available normal human bronchus sections from 3 independent donors for 6 of the aging signature genes whose expression are dysregulated by smoking: C3, clusterin (CLU), CX3CL1, LAPTM5, MGST1 and SPP1. The staining results clearly demonstrate that all 6 genes are epithelial expressed (Figure 3). Overall, these data demonstrate that smoking results in significant abnormal expression of a number of aging-related genes in the small airway epithelium.

**Table 3:** **Aging genes differentially expressed in the small airway epithelium of healthy smokers** ***vs*** **healthy nonsmokers** ^**1**^

**Direction of expression with smoking**	**Gene symbol**	**Gene title**	**Fold-change** ^**2**^	**p value** ^**3**^	**Direction of expression with aging** ^**4**^
Up-regulated	SPP1	Secreted phosphoprotein 1	13.48	3.1x10^−5^	Over
	LAPTM5	Lysosomal protein transmembrane 5	1.75	3.8x10^−2^	Over
	ANXA3	Annexin A3	1.72	2.2x10^−3^	Over
	MPEG1	Macrophage expressed 1	1.59	1.3x10^−2^	Over
	MGST1	Microsomal glutathione S-transferase 1	1.40	2.4x10^−4^	Over
	MSN	Moesin	1.39	2.6x10^−2^	Over
	CLU	Clusterin	1.38	3.6x10^−3^	Over
	COL3A1	Collagen, type III, alpha 1	1.34	4.3x10^−2^	Under
	FCGR2B	Fc fragment of IgG, low affinity IIb, receptor (CD32)	1.32	3.4x10^−2^	Over
	GSTA1	Glutathione S-transferase alpha 1	1.31	3.8x10^−4^	Over
	LITAF	Lipopolysaccharide-induced TNF factor	1.26	3.2x10^−4^	Over
	GHITM	Growth hormone inducible transmembrane protein	1.25	3.1x10^−5^	Under
	VAT1	Vesicle amine transport protein 1 homolog (T. californica)	1.22	4.3x10^−3^	Over
Down-regulated	MT1F	Metallothionein 1 F	−1.60	3.1x10^−5^	Over
	PCSK6	Proprotein convertase subtilisin/kexin type 6	−1.63	4.7x10^−4^	Over
	C4A	Complement component 4A	−2.45	3.2x10^−4^	Under
	CX3CL1	Chemokine (C-X3-C motif) ligand 1	−2.50	3.0x10^−6^	Under
	C3	Complement component 3	−2.73	2.1x10^−3^	Over

^1^List of aging genes based on de Magalhaes et al. [26]; listed are the genes with significantly different expression in the small airway epithelium of healthy smokers *vs* healthy nonsmokers; for the data for all of the de Magalhaes et al. “aging genes”, see Additional file 1: Table S1.

^2^Fold-change - mean in healthy smokers/mean in healthy nonsmokers.

^3^False discovery rate, p < 0.05, Partek Benjamini-Hochberg correction.

^4^Direction of expression observed with aging, see de Magalhaes et al. [26].

### Effect of smoking on telomere length in the small airway epithelium

**Figure 4:**
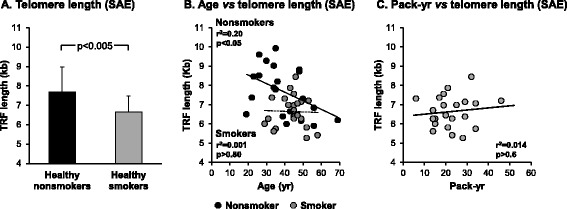
**Telomere length in the small airway epithelium of nonsmokers and smokers**. DNA was isolated from the small airway epithelium of male healthy nonsmokers (n = 21) and male healthy smokers (n = 22) and telomere length [terminal restriction fragment (TRF)] quantified by Southern analysis. **A**. Data shown is represented as the average ± standard deviation of the TRF length (kb). The difference in mean TRF length between phenotypes was calculated by 2-tailed Students t test. **B**. Correlation of telomere length with age. Telomere length was quantified by Southern analysis and correlated with the age of each individual subject. Correlations between mean telomere length and age were performed using linear regression. Black circles represent healthy nonsmokers and grey circles represent healthy smokers. **C**. Correlation of telomere length with smoking history (pack-yr). Telomere length from healthy smokers was quantified by Southern analysis and correlated with the smoking history for each individual subject. Correlations between mean telomere length and smoking history were performed using linear regression.

The effect of smoking on aging of the small airway epithelium was assessed using telomere length analysis as an alternative marker of aging in an independent cohort of age-matched healthy nonsmokers and smokers (Table 2). Because of the known differences in telomere length between males and females [29] we restricted the analysis to only males. Telomere length of DNA extracted from the small airway epithelium was assessed by Southern analysis of *HinfI* and *RsaI* digested total DNA and the mean terminal restriction fragment (TRF) length calculated for each sample. In healthy nonsmokers (n = 21) the mean TRF length was 7.69 ± 1.41 kb whereas in age-matched healthy smokers (n = 22), the mean TRF length was 6.65 ± 1.16 kb, and significantly shorter (p < 0.005; Figure 4A). As expected, in the healthy nonsmokers, telomere length significantly decreased with age (r^2^ = 0.20, p < 0.05; Figure 4B). In contrast, in healthy smokers there was no significant decrease in telomere length with age (r^2^ = 0.001, p > 0.8). Telomere length in smokers was not dependent on smoking history, with no correlation between telomere length and pack-yr (r^2^ = 0.014, p > 0.6; Figure 4C). To assess the effect of race on smoking dependent telomere shortening in the small airway epithelium, we compared telomere length in the small airway epithelium of smokers *vs* nonsmokers subdivided by racial categories (Black, White and Other). The results demonstrate that in each racial subgroup there is a trend of shorter telomeres in smokers *vs* nonsmokers (Black, 6.77 ± 0.92 kb *vs* 7.61 ± 1.28 kb; White, 5.88 ± 0.35 kb *vs* 7.57 ± 1.32 kb and Other, 6.77 ± 0.55 kb *vs* 7.86 ± 1.55 kb; Additional file 1: Figure S2). Due to the small sample size for each subgroup statistical significance in telomere length differences is only observed between White smokers *vs* nonsmokers (p < 0.03), however due to consistency of the smoking dependent effects on telomere length between racial subgroups the data suggests that race plays no significant role in smoking dependent telomere shortening of the small airway epithelium.

Previous studies have shown that telomere length is shorter in peripheral blood leukocytes of smokers compared to nonsmokers [6-18]. Cell differential analysis of the small airway epithelial brushes of nonsmokers and smokers demonstrated epithelial cells were the dominant cell population with a small amount of inflammatory cells. However, there was a significantly lower level of inflammatory cells in the brushings of smokers compared to nonsmokers (0.9% *vs* 2.5%, p < 0.0001; Table 2) suggesting the reduced telomere length we observe in the small epithelium of smokers does not result from the presence of inflammatory cells and are specific for the epithelial cell populations. In support of this, we measured the telomere length in the peripheral blood leukocytes of a subset of age matched nonsmokers (n = 12/21) and smokers (n = 16/22) (40.8 ± 9.7 yrs nonsmokers and 46 ± 6.4 yrs smokers, p > 0.12). The results demonstrated no significant differences (p > 0.1) in the telomere length of peripheral blood leukocytes in smokers *vs* nonsmokers (7.04 ± 0.71 kb *vs* 7.42 ± 0.54 kb; Additional file 1: Figure S3). However, in the same samples we still observe a significant decrease (p < 0.002) in the telomere length of the small airway epithelium in smokers *vs* nonsmokers (6.49 ± 0.74 kb *vs* 7.95 ± 1.26 kb). Therefore, these data suggest that reduced telomere length observed in the small epithelium of smokers is specific for the epithelial cell populations. Overall, these data suggest that smoking prematurely ages the small airway epithelium with smokers displaying on average telomere lengths ~14% shorter than chronologically age-matched healthy nonsmokers.

## Discussion

During the natural aging process of healthy individuals, the lung undergoes a progressive decrease in lung function [3,4]. Studies have demonstrated that cigarette smoking accelerates the rate of lung function decline, suggesting that smoking results in premature aging of the lung [3,5]. The small airway epithelium consists of 4 major cell types that line the airway lumen ≥6th generations and take the initial brunt of inhaled toxins from the cigarette smoke [20,21,24]. The earliest abnormalities and pathology linked to smoking and development of COPD manifest in the small airway epithelium [19,22,23], and biologic changes in the small airway epithelium in response to cigarette smoking can be observed long before the presence of pathology [25,27,30]. The focus of this study was to determine if cigarette smoking results in accelerated aging of the small airway epithelium at the molecular level before physical manifestation of lung disease or lung function decline. To address this question we used the sensitive molecular parameters of aging-related gene expression and telomere length to compare the aging process of the small airway epithelium in age matched healthy nonsmokers and healthy smokers. The data demonstrate that smoking significantly dysregulates expression of an aging gene signature in the small airway epithelium, with ~26% of the aging genes (18/67) up or down-regulated greater than 1.2-fold in response to smoking. Over 72% (13/18) of the 18 smoking dysregulated genes demonstrate the same direction of expression observed with aging, suggesting smoking is accelerating the aging process in the small airway epithelium. In support of this concept, telomere length analysis in the small airway epithelial cells of an independent cohort of age matched male subjects revealed smoking status had a significant impact on telomere length, with smokers displaying on average a ~14% decrease in telomere length compared to nonsmokers. One of the limitations of this study is the presence of multiple cell types and epithelial subsets in the brushings obtained during bronchoscopy which makes it difficult to determine the individual cell type responsible for the accelerated aging in response to smoking. However, the epithelial brushes we analyze are processed immediately upon collection thus reducing any potential artifacts from downstream processing and thus represent a true reflection of the *in vivo* environment. In addition, the differences in number of epithelial cell subsets (ciliated, secretory, basal and undifferentiated columnar) between nonsmokers and smokers may allow inference towards the cell populations responsible for the smoking dependent changes we observe at the gene expression level and in telomere length and suggest that specific cell populations are more susceptible to the aging effects of cigarette smoke. Currently the biological consequences of smoking-dependent changes in age related gene expression and telomere shortening in the small airway epithelium on lung function decline and/or clinical disease in smokers are unknown. In addition, it is currently unknown if the effects of aging gene dysregulation and telomere shortening are mutually exclusive with each contributing independently to development of specific lung disease (e.g. COPD *vs* lung cancer) or both processes are biologically connected and contribute equally to development of disease. Future studies dissecting the biological consequences of each molecular phenomenon will help clarify their role in development of specific lung diseases. However, based on the known function of the aging signature genes and telomeres we can postulate potential consequences of these changes.

### Consequence of aging-related gene expression changes in the small airway epithelium

Characterization of the biological processes associated with the original 73 gene aging signature identified by de Magalhaes et al. [26] highlighted enrichment for genes involved in multiple cellular processes, including immune response, inflammation, mitochondrial function, metabolism, lysosomes, collagen, apoptosis, cell cycle and markers of cellular senescence. Pathway analysis of the 18 smoking dysregulated genes failed to identify enrichment of a single biological process. However, based on the known biological functions of a subset of these genes, we can hypothesize how they may play a role in regulating the response of the small airway epithelium to cigarette smoke exposure and their potential contribution to the development of long-term pathological changes associated with smoking.

Based on the hypothesis that age-related gene expression changes may have a protective role in mediating the harmful effects of cellular dysfunction over-time, smoking-induced expression of these genes may serve a similar role. For instance, increased expression of the anti-oxidant genes MGST1 and GSTA1 in smokers most likely protects against elevated levels of harmful oxidants [31-33]. In addition, elevated expression of the protein chaperone clusterin (CLU) may curtail the effects of protein mis-folding and aggregation in response to cigarette smoke [34,35].

Alternatively, dysregulated expression of some genes may play a key role in driving the pathological changes associated with cigarette smoking. SPP1, the most highly induced smoking-responsive aging gene, encodes osteopontin, a secreted cytokine-like molecule that mediates immune function and extracellular matrix (ECM) remodeling [36]. A recent study demonstrated that cigarette smoke-dependent up-regulation of SPP1 expression mediates induction of emphysema in an experimental mouse model [37]. In addition, asbestos exposure in a murine lung fibrosis model results in up-regulation of SPP1 in bronchiolar epithelial cells [38]. Microarray analysis comparing lungs of wild-type and SPP1 knockout mice exposed to asbestos showed that SPP1 modulated expression of a number of genes involved in cell signaling, immune system/defense, cell cycle regulation and ECM remodeling [38]. Therefore, elevated expression of SPP1 in the small airway epithelium of smokers may have significant long term pathological consequences and may play a key role in the process of airway remodeling characterized in smoking-induced respiratory disease such as COPD.

While a majority of the smoking dysregulated aging genes (13/18) displayed the same direction of smoking-induced expression as that observed with aging, the remaining 5 genes (COL3A1, GHITM, MT1F, PCSK6 and C3) demonstrated the opposite direction of expression observed with aging. At present, the long-term biological consequence of these alternate expression patterns is unknown. However, studies for COL3A1, which encodes for collagen 3A1, have shown that expression of this gene is positively regulated by SPP1 in a murine model of asbestos-induced lung fibrosis and that over-expression of the gene is linked to fibrosis and tissue remodeling [38]. Therefore, while COL3A1 expression generally decreases with aging over time to possibly reduce age-related tissue remodeling, smoking-induced expression may contribute significantly to the increased incidence of airway remodeling in individuals with smoking-induced respiratory disease such as COPD [19,22,23,39,40]. Similarly to COL3A1, the putative tumor suppressor gene MT1F may contribute to smoking-induced pathological changes. MT1F expression is typically down-regulated in a majority of human colon tumor tissues and its enforced expression can result in increased apoptosis and inhibition of cell migration, invasion and *in vivo* tumorigenicity [41]. Therefore, the general increase in expression of MT1F during aging may function as a protective mechanism to prevent development of cancer, whereas smoking-dependent down-regulation in the small airway epithelium may contribute to the increased risk of developing lung cancer in smokers [42]. However, additional *in vitro* studies need to be performed to demonstrate a role of MT1F in development of lung cancer in addition to following subjects over time to confirm the correlation between MT1F expression and development of lung cancer and/or COPD.

### Consequence of telomere length changes in the small airway epithelium

Based on the hypothesis that COPD is a disease of accelerated aging [43-46], a number of different studies have analyzed the effect of smoking and COPD status on telomere length in blood leukocytes [6-18]. While some have demonstrated that smokers (independent of COPD status) have shorter blood leukocyte telomeres compared to nonsmokers [6,7,10-13], others have shown that COPD status, but not smoking is associated with shortened telomeres [15-18]. Recent findings from a large study group of 46,396 individuals from the Danish general population demonstrate that blood leukocyte short telomere length is associated with decreased lung function and increased risk of COPD [8]. Furthermore, a meta-analysis of blood leukocyte telomere length and COPD from nine independent European studies demonstrated shorter telomere length in COPD subjects compared with healthy controls [14]. Our study is the first to look at the effect of smoking on telomere length in the small airway epithelium and demonstrates that smoking accelerates telomere shortening in this cell population critical to lung function prior to the development of lung disease and lung function decline. In addition, our analysis of telomere length in the peripheral blood leukocytes in a subset of the nonsmokers and smokers in our study suggest the small airway epithelium is more sensitive to the telomere shortening effects of cigarette smoke exposure than peripheral blood leukocytes. In contrast to studies of blood leukocyte telomere length, we observed no negative correlation of telomere length with pack-yr. In our analysis we did not correct for confounders such as age and obesity as no significant differences are observed in these parameters between our nonsmoker and smoker groups. The presence of additional confounders may explain the lack of negative association of telomere length with pack-yr; alternatively it may result from the narrow range of pack-yr of our subjects which may prevent identification of an association. In addition, from a biological perspective a small amount of cigarette smoke exposure may result in significant effects on telomere length, therefore, masking any effect between individuals with medium to high pack-yr. The long term biological consequences of smoking-dependent telomere shortening in the small airway epithelium are unknown, however based on the function of telomeres, one may postulate a number of possible outcomes.

Telomeres are specialized regions at the ends of linear chromosomes comprised of TTAGGG repeats that play a key role in maintaining chromosome stability and are linked to aging [47]. In germ cells, telomeres are maintained by the enzyme complex telomerase; however, lack of expression of telomerase in somatic tissues results in decreased telomere length following each round of cell division [47]. Once telomere length shortens to a specific point, a DNA damage response is elicited that is largely p53-dependent, resulting in reduced replication potential of the cell and activation of senescence with subsequent removal of the cells by apoptosis [48-51]. The numbers of senescent cells are known to increase in tissues during the natural aging process either due to an increase in their generation or decrease in their removal [2]. In contrast to normal cells, senescent cells are known to adopt an altered phenotype highlighted by loss of proliferative activity, persistence of active metabolism, altered secretome (enriched in pro-inflammatory cytokines and matrix metalloproteinases) and resistance to apoptosis which can lead to loss of tissue homeostasis [49,51,52]. Therefore, smoking-dependent accelerated telomere shortening may increase the rate at which cells of the small airway epithelium reach cell senescence. In support of this concept, *in vivo* studies have shown that alveolar epithelial cells from COPD smokers have increased numbers of senescent cells relative to healthy controls [53,54]. Furthermore, during normal aging increased expression of the smoking-induced aging gene CLU is used as a marker for cell senescence [55]. Therefore, our gene expression data supports the hypothesis that telomere shortening may increase the rate of senescence in the small airway epithelium. In addition, studies of lung endothelial cells from COPD smokers demonstrated they reached senescence earlier during *in vitro* culture relative to nonsmokers and in doing so, adopted an abnormal secretory phenotype with secretion of a diverse array of cytokines known to play a role in inflammation and airway remodeling [56]. Senescent cells within the small airway may adopt a similar function and secrete mediators that further drive smoking-induced pathology and airway remodeling.

An alternative consequence of shorter telomere length in the small airway epithelium maybe a decline in resident stem/progenitor cell function similar to what is observed in other aging tissues [2,47]. Basal cells are the stem/progenitor population of cells in the small airways that differentiate into the other specialized epithelial cell types of the airway during normal epithelial turnover and repair [57-59]. *In vitro* studies have demonstrated that continuous culture of these cells to a senescent state results in diminished differentiation capacity [60,61]. Therefore, reduced telomere length in the airway basal cells of smokers may result in impaired differentiation capacity leading to disordered cell differentiation and airway epithelial remodeling [58]. In support of this, the first manifestation of smoking-induced pathology *in vivo* of the small airway epithelium is basal cell hyperplasia which is characterized by increased proliferation and expansion of the basal cell population [19]. However, at present we have no evidence suggesting telomere length in the basal cell population is reduced in response to cigarette smoke exposure. Future studies identifying the exact cell populations of the small airway epithelium that are more sensitive to smoking-dependent telomere shortening will help further our understanding of the biological consequences of this phenomenon.

To date, the evidence clearly shows that short telomere length is associated with incidence of specific lung diseases; however, it is unknown whether telomere shortening itself drives the pathological processes in the lung or whether this phenomenon itself is a bystander and just a consequence of other processes at work. A recent study using telomerase null mice with short telomeres demonstrated that short telomeres alone was not sufficient to induce spontaneous lung disease; however, when mice with short telomeres were experimentally exposed to cigarette smoke, they had a lower threshold to develop emphysema [62]. These findings suggest that multiple factors contribute to the development of cigarette smoke-induced airway disease and that telomere length, at least in part, plays a partial active role in “sensitizing” the cells to further damage and destruction. Therefore, similar mechanisms may be conserved in humans, and individuals with short telomeres may be of increased risk for development of smoking-induced COPD.

## Conclusion

In summary, these data provide evidence that smoking accelerates aging of the small airway epithelium at the molecular level. Future studies analyzing the function of smoking-induced aging-related genes and telomeres in small airway epithelium biology may help identify new therapeutic targets to treat smoking-induced lung disease.

## Additional file

Additional file 1:
**Supplemental Data.**
