# Supplementary material for: Smoking accelerates aging of the small airway epithelium
Source: Respir Res. 2014 Sep 24;15(1):94. doi: 10.1186/s12931-014-0094-1 (PMC4189169; doi:10.1186/s12931-014-0094-1)

Supplemental Methods

Inclusion and Exclusion Criteria

Healthy nonsmokers

Inclusion criteria

- Males and females, at least 18 years old
- Provide informed consent
- Normal physical examination
- HIV1 negative
- Not pregnant (females)
- Good health without history of chronic lung disease, including asthma, and without recurrent or recent (within 3 months) acute pulmonary disease
- Normal routine laboratory evaluation, including general hematologic studies, general serologic/immunologic studies, general biochemical analyses, and urine analysis
- Normal PA and lateral chest X-ray
- Normal electrocardiogram (sinus bradycardia, premature atrial contractions are permissible)
- No history of allergies to medications used in the bronchoscopy procedure
- Not taking any medications relevant to lung disease or having an effect on the airway epithelium
- Willingness to participate in the study
- Self-reported nonsmokers, with smoking status validated by the absence of nicotine and cotinine in urine (nicotine<2 ng/ml, cotinine<5 ng/ml)

Exclusion criteria

- Unable to meet the inclusion criteria
- Current active infection or acute illness of any kind
- Evidence of malignancy within the past 5 years
- Alcohol or drug abuse within the past 6 months

Healthy active smokers

Inclusion criteria

- Males and females, at least 18 years old
- Provide informed consent
- Normal physical examination
- HIV1 negative
- Not pregnant (females)
- Good health without history of chronic lung disease, including asthma, and without recurrent or recent (within 3 months) acute pulmonary disease
- Normal routine laboratory evaluation, including general hematologic studies, general serologic/immunologic studies, general biochemical analyses, and urine analysis
- Normal PA and lateral chest X-ray
- Normal electrocardiogram (sinus bradycardia, premature atrial contractions are permissible)
- No history of allergies to medications used in the bronchoscopy procedure

- Not taking any medications relevant to lung disease or having an effect on the airway epithelium
- Willingness to participate in the study
- Self-reported current daily smokers with any number of pack-yr, validated by urine nicotine ≥30 ng/ml and cotinine ≥50 ng/ml

Exclusion criteria

- Unable to meet the inclusion criteria
- Current active infection or acute illness of any kind
- Evidence of malignancy within the past 5 years
- Alcohol or drug abuse within the past 6 months

References

1. de Magalhaes JP, Curado J, Church GM: **Meta-analysis of age-related gene expression profiles identifies common signatures of aging.** *Bioinformatics* 2009, **25:**875-881.

Supplemental Table I. List of Aging Signature Genes Expressed in Small Airway Epithelium[1,2]

Probeset ID[3]	Gene symbol	Gene title
234312_s_at	ACSS2	acyl-CoA synthetase short-chain family member 2
201346_at	ADIPOR2	adiponectin receptor 2
209369_at	ANXA3	annexin A3
200782_at	ANXA5	annexin A5
201525_at	APOD	apolipoprotein D
207508_at	ATP5G3	ATP synthase; H+ transporting; mitochondrial Fo complex; subunit C3
201891_s_at	B2M	beta-2-microglobulin
218232_at	C1QA	complement component 1; q subcomponent; A chain
202953_at	C1QB	complement component 1; q subcomponent; B chain
225353_s_at	C1QC	complement component 1; q subcomponent; C chain
217767_at	C3	complement component 3
214428_x_at	C4A	complement component 4A (Rodgers blood group)
201310_s_at	C5orf13	chromosome 5 open reading frame 13
206209_s_at	CA4	carbonic anhydrase IV
205626_s_at	CALB1	calbindin 1; 28kDa
201560_at	CLIC4	chloride intracellular channel 4
208791_at	CLU	clusterin
1556499_s_at	COL1A1	collagen; type I; alpha 1
215076_s_at	COL3A1	collagen; type III; alpha 1
213110_s_at	COL4A5	collagen; type IV; alpha 5
202902_s_at	CTSS	cathepsin S
823_at	CX3CL1	chemokine (C-X3-C motif) ligand 1
218172_s_at	DERL1	Der1-like domain family; member 1
219350_s_at	DIABLO	diablo homolog (Drosophila)
201842_s_at	EFEMP1	EGF-containing fibulin-like extracellular matrix protein 1
205738_s_at	FABP3	fatty acid binding protein 3
203561_at	FCGR2A	Fc fragment of IgG; low affinity IIa; receptor (CD32)
210889_s_at	FCGR2B	Fc fragment of IgG; low affinity IIb; receptor (CD32)
202748_at	GBP2	guanylate binding protein 2; interferon-inducible
209249_s_at	GHITM	growth hormone inducible transmembrane protein
212335_at	GNS	glucosamine (N-acetyl)-6-sulfatase
201141_at	GPNMB	glycoprotein (transmembrane) nmb
203924_at	GSTA1	glutathione S-transferase alpha 1
209458_x_at	HBA1 /// HBA2	hemoglobin; alpha 1 /// hemoglobin; alpha 2
223640_at	HCST	hematopoietic cell signal transducer
209398_at	HIST1H1C	histone cluster 1; H1c
211530_x_at	HLA-G	major histocompatibility complex; class I; G
212592_at	IGJ	immunoglobulin J polypeptide
209821_at	IL33	interleukin 33
201776_s_at	KIAA0494	KIAA0494
201720_s_at	LAPTM5	lysosomal protein transmembrane 5
208949_s_at	LGALS3	lectin; galactoside-binding; soluble; 3
200704_at	LITAF	lipopolysaccharide-induced TNF factor
1555745_a_at	LYZ	lysozyme
224918_x_at	MGST1	microsomal glutathione S-transferase 1
226818_at	MPEG1	macrophage expressed 1
200600_at	MSN	moesin
213629_x_at	MT1F	metallothionein 1F

Supplemental Table I. List of Aging Signature Genes Expressed in Small Airway Epithelium[1,2] (cont., page 2)

Probeset ID[3]	Gene symbol	Gene title
200632_s_at	NDRG1	N-myc downstream regulated 1
218320_s_at	NDUFB11	NADH dehydrogenase (ubiquinone) 1 beta subcomplex; 11
200701_at	NPC2	Niemann-Pick disease; type C2
207414_s_at	PCSK6	proprotein convertase subtilisin/kexin type 6
208777_s_at	PSMD11	proteasome (prosome; macropain) 26S subunit; non-ATPase; 11
200627_at	PTGES3	prostaglandin E synthase 3 (cytosolic)
225562_at	RASA3	RAS p21 protein activator 3
225931_s_at	RNF213	ring finger protein 213
203186_s_at	S100A4	S100 calcium binding protein A4
217728_at	S100A6	S100 calcium binding protein A6
200986_at	SERPING1	serpin peptidase inhibitor; clade G (C1 inhibitor); member 1
201739_at	SGK1	serum/glucocorticoid regulated kinase 1
209875_s_at	SPP1	secreted phosphoprotein 1
207332_s_at	TFRC	transferrin receptor (p90; CD71)
212352_s_at	TMED10	transmembrane emp24-like trafficking protein 10 (yeast)
201008_s_at	TXNIP	thioredoxin interacting protein
208909_at	UQCRFS1	ubiquinol-cytochrome c reductase; Rieske iron-sulfur polypeptide 1
201568_at	UQCRQ	ubiquinol-cytochrome c reductase; complex III subunit VII; 9.5kDa
208626_s_at	VAT1	vesicle amine transport protein 1 homolog (T. californica)

[1] 73 gene aging signature was taken from de Magalhaes et al 2009.

[2] 67 genes of the published 73 gene aging signature contained U133 2.0 probesets and were expressed in the small airway epithelium of healthy nonsmokers (P call ≥10).

[3] Affymatrix U133 2.0 microarray.

Supplemental Figure Legends

Supplementary Figure 1. Southern analysis of telomere length in the small airway epithelium and peripheral blood leukocytes of nonsmokers and smokers. Telomere length [terminal restriction fragment (TRF)] of DNA isolated from the small airway epithelium and blood of male healthy nonsmokers and male healthy smokers was quantified by Southern blot analysis. **A.** Representative blot from small airway epithelium (SAE) samples. **B.** Representative blot from blood leukocyte samples. For both **A** and **B** lanes 1-3 represent 3 independent nonsmoker samples and lanes 4-6 represent 3 independent smoker samples.

Supplementary Figure 2. Telomere length in the small airway epithelium of nonsmokers and smokers from different racial categories. DNA was isolated from the small airway epithelium of male healthy nonsmokers (n=8 Black, n=6 White, and n=7 Other) and male healthy smokers (n=14 Black, n=3 White and n=5 Other) and telomere length [terminal restriction fragment (TRF)] quantified by Southern analysis. Within each phenotype the telomere length of subjects was separated based on racial group. Data shown is represented as the average ± standard deviation of the TRF length (kb). The difference in mean TRF length between phenotypes was calculated by 2-tailed Students t test.

Supplementary Figure 3. Telomere length in the small airway epithelium and peripheral blood leukocytes of nonsmokers and smokers. DNA was isolated from the small airway epithelium and peripheral blood leukocytes of male healthy nonsmokers (n=12) and male healthy smokers (n=16) and telomere length [terminal restriction fragment (TRF)] quantified by Southern analysis. Data shown is represented as the average ± standard deviation of the TRF length (kb). The difference in mean TRF length between phenotypes was calculated by 2-tailed Students t test.

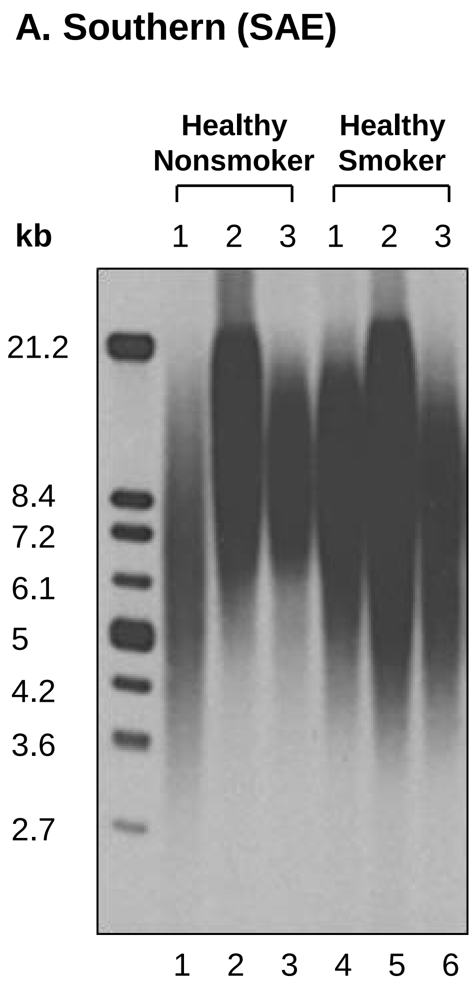
A. Southern (SAE)
Healthy Nonsmoker
Healthy Smoker
kb
1 2 3 1 2 3
21.2
8.4
7.2
6.1
5
4.2
3.6
2.7
1 2 3 4 5 6

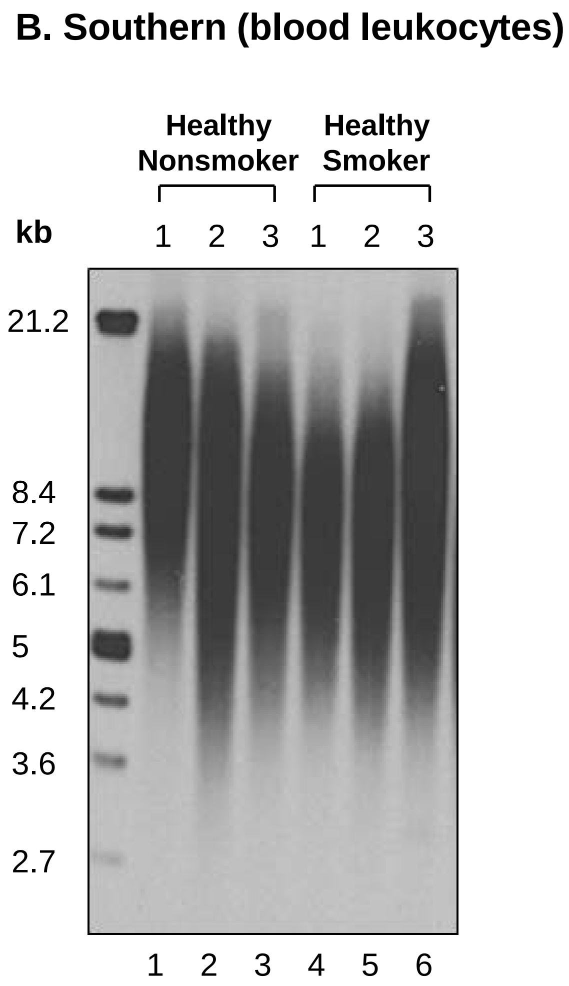
B. Southern (blood leukocytes)
Healthy Nonsmoker
Healthy Smoker
kb
1 2 3 1 2 3
21.2
8.4
7.2
6.1
5
4.2
3.6
2.7
1 2 3 4 5 6

Supplementary Figure 2

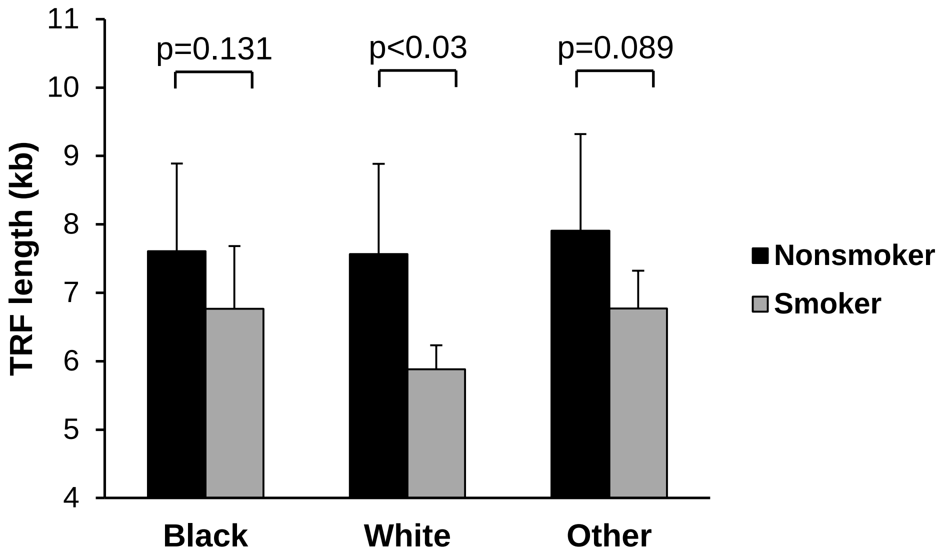

Supplementary Figure 3

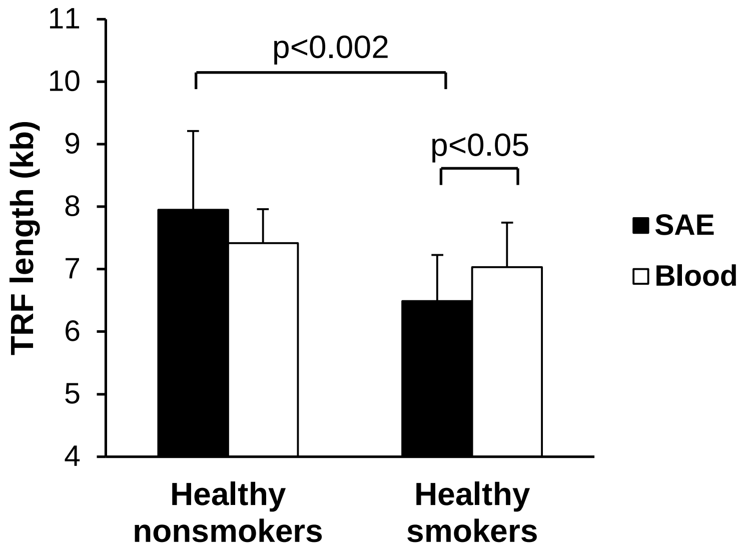

Supplement: Additional file 1: — Supplemental Data. [file 12931_2014_94_MOESM1_ESM.pdf]
